# Oral *Brucella melitensis* infection leads to persistent bacterial colonisation and dynamic histopathology in reproductive and immune organs of female mice

**DOI:** 10.3389/fmicb.2026.1871530

**Published:** 2026-07-06

**Authors:** Chuang Li, Jiarui Luo, Zhenglong Chai, Siyu Zhang, Xuanji Zhang, Zhendong Zhang, Yuejie Zhu, Fengbo Zhang

**Affiliations:** 1 Department of Clinical Laboratory, The First Affiliated Hospital of Xinjiang Medical University, Urumqi, Xinjiang, China; 2 Department of Orthopedic Surgery, The First Affiliated Hospital of Shihezi University, Shihezi, China; 3 Reproductive Medicine Center, The First Affiliated Hospital of Xinjiang Medical University, Urumqi, Xinjiang, China

**Keywords:** *Brucella*, flow cytometry, immunohistochemistry, morphology, oral infection, pathological injury, spleen, uterus

## Abstract

Oral infection is a major route of *Brucella* transmission; however, systematic reports on the dynamic multiorgan damage induced by this route are still lacking. *Brucella* preferentially colonises reproductive organs (e.g., the uterus) and immune organs, leading to reproductive system diseases and chronic infection in humans. In this study, female BALB/c mice were inoculated with *Brucella melitensis* (*B. melitensis*) via intragastric gavage. Uterus, spleen, lung, and lymph node samples were collected over 8 weeks post-infection, and bacterial burden, organ weight, H&E histopathology, immunohistochemical staining for *Brucella* antigen, and flow cytometric analysis of F4/80^+^CD11b^+^ macrophages were evaluated. The results showed that bacteria were detectable in all examined organs as early as 1 day post-infection, indicating rapid dissemination from the gastrointestinal tract. Bacterial loads in the lung and lymph nodes peaked at 1 week and then declined, but remained detectable up to 8 weeks. In the spleen and uterus, bacterial loads peaked at 2 weeks, and the bacterial load in the reproductive organ (uterus) was consistently lower than that in immune organs (spleen and lymph nodes). Splenic weight peaked at 2 weeks, whereas uterine weight peaked at 4 weeks. Histopathology revealed that the most severe lesions in the uterus, spleen, and lymph nodes occurred at 2 weeks post-infection, with bacteria persisting up to 8 weeks. The lung exhibited acute lesions followed by marked inflammatory resolution, with a significant reduction in bacterial load at 8 weeks. Flow cytometry showed that the proportion of F4/80^+^CD11b^+^ macrophages in the uterus and spleen increased significantly from 1 to 4 weeks post-infection, peaked at 2 weeks, and gradually decreased to levels below those of the PBS control group by 8 weeks (*p*<0.01). This study systematically describes the dynamic pathological and immunological changes in the reproductive and immune organs of mice following oral *Brucella* infection, providing a mouse model basis for studies on the pathogenesis of brucellosis and vaccine evaluation.

## Introduction

Brucellosis is a globally distributed zoonotic infectious disease caused by bacteria of the genus *Brucella* (Djokic et al., 2023; Li et al., 2025). Oral transmission is one of the main routes of infection in humans and animals, occurring primarily through the consumption of contaminated food or water (e.g., unpasteurised dairy products) (Tian et al., 2024; Chai et al., 2025; Ghssein et al., 2025). *Brucella* has a propensity to invade and colonise reproductive and immune organs, leading to chronic infection and reproductive system diseases in humans, such as endometritis; long-term infection can involve the myometrium, causing myositis and abnormal uterine contractions, which further aggravate adverse pregnancy outcomes (Sebzda and Kauffman, 2023; Shi et al., 2025). However, the dynamic multi-organ damage (especially to female reproductive organs and immune organs) caused by the oral infection route has not yet been systematically reported in mice (Hensel et al., 2020).

The mouse model is widely used in basic research on brucellosis because of its low cost, well-defined genetic background, and abundant immunological reagents (Osman et al., 2018; Ghssein et al., 2025). *Brucella* is a facultative intracellular bacterium that, upon entering the host, primarily colonises and replicates within macrophages (Dugelay et al., 2025). Studies have shown that the peak of *Brucella* infection is closely associated with the formation of multinucleated giant cell granulomas, which are composed mainly of CD11b^+^F4/80^+^MHC-II^+^ cells and express iNOS/NOS2. As an important immune barrier restricting *Brucella* dissemination, granuloma formation depends on the recruitment and aggregation of such activated monocyte/macrophage populations (Copin et al., 2012). Regarding oral immunisation research, in 1990, investigators used an attenuated Salmonella vector to induce serum and intestinal antibodies against *Brucella* antigens in mice via the oral route (Stabel et al., 1990). The biological significance of the oral route in *Brucella* infection and immunity is not limited to mouse models. In large animals such as cattle, oral vaccination with the RB51 vaccine strain has also been shown to significantly reduce post-infection abortion rates and bacterial colonisation, without producing O-polysaccharide antibodies that interfere with serological diagnosis, indicating that oral immunisation is feasible in large animals and holds promising clinical prospects (Elzer et al., 1998). Nevertheless, a longitudinal description of bacterial colonisation kinetics, histopathological evolution, and local immune cell (e.g., macrophage subset) dynamics in multiple organs (especially reproductive and immune organs) following oral challenge is still lacking.

In existing studies, the routes of infection are mostly intraperitoneal injection, aerosol inhalation, or conjunctival inoculation, while the oral intragastric route is less frequently used (Hielpos et al., 2017; Sharif et al., 2024). Given the epidemiological importance of oral transmission in human brucellosis, establishing a well-characterised mouse model of oral infection and systematically describing the bacterial colonisation kinetics, histopathological evolution, and local macrophage dynamics in multiple organs is of significance for understanding the pathogenesis of brucellosis and evaluating vaccine efficacy. Therefore, this study aims to systematically investigate the bacterial colonisation kinetics, histopathological evolution, immunohistochemical features, and dynamic changes of F4/80^+^CD11b^+^ macrophages in multiple organs (uterus, spleen, lung, and lymph nodes) of female BALB/c mice after intragastric inoculation with *B. melitensis*.

We hypothesise that oral infection leads to rapid dissemination of *Brucella* throughout the body, with distinct colonisation and pathological kinetics in different organs; the patterns of damage in reproductive organs and immune organs may differ, and macrophages play a key role in the local immune response. By covering multiple time points from 1 day to 8 weeks post-infection and using a combination of bacterial culture, organ weighing, histopathology, immunohistochemistry, and flow cytometry. The results will provide a novel animal model basis for the pathogenesis of brucellosis and serve as a reference for future evaluation of vaccines and drugs.

## Materials and methods

### Ethics statement

All animal experimental protocols involved in this study were approved by the Medical Ethics Committee of the First Affiliated Hospital of Xinjiang Medical University (affiliated to the Medical Experimental Animal Ethics Committee of the Xinjiang Uygur Autonomous Region), and all procedures complied with China’s national regulations for animal protection. All experimental procedures adhered to the relevant regulations for the care and use of laboratory animals and were conducted in a Biosafety Level 3 (BSL-3) laboratory. Experiments involving live *Brucella* were performed in the BSL-3 laboratory of the Xinjiang Uygur Autonomous Region Center for Disease Control and Prevention (Xinjiang CDC). This laboratory meets the national biosafety standards CNAS-CL05:2009 and GB 19489:2008 and has obtained accreditation for BSL-3 operations from the China National Accreditation Service for Conformity Assessment (CNAS) (Registration No. CNAS BL0099). The facility is equipped with negative-pressure ventilation, HEPA-filtered air handling systems, and dedicated Animal Biosafety Level 3 (ABSL-3) housing. All operators followed BSL-3 laboratory practices and wore personal protective equipment throughout all procedures. *Brucella* culture and bacterial suspension preparation were performed in a Type A2 biological safety cabinet. To prevent aerosol generation, oral gavage inoculation was also conducted inside a biological safety cabinet. Infected animals were housed in individually ventilated cages with HEPA filtration within the ABSL-3 facility throughout the infection period.

### Experimental animals

A total of 100 specific pathogen-free (SPF) female BALB/c mice, aged 6–8 weeks and weighing 18–22 g, were purchased from the Animal Experimental Center of Xinjiang Medical University. All mice were housed in isolator cages within a BSL-3 laboratory, with 3–4 animals per cage, under controlled conditions of temperature at 22°C ± 1°C and relative humidity at 50% ± 5%, and maintained on a 12h light/12h dark cycle (Silva-Santana et al., 2020). Prior to infection, the mice were acclimatised for 1 week; during the acclimation period and throughout the experiment, food and water were provided *ad libitum*. The general physiological status of the mice was observed daily, and mice were excluded if they fell outside the specified body weight range, showed visible injuries, exhibited abnormal behaviour, presented signs of illness, or had poor overall condition.

### Bacterial strain and preparation of bacterial suspension

The *Brucella* melitensis strain 16M (*B. melitensis 16M*) (ATCC 23456) was purchased from the China Institute of Veterinary Drug Control (IVDC; National Center for Veterinary Microbial Cultures, CVCC), Beijing, China. The standard strain *B. melitensis 16M* was used in this study. The strain was preserved at –80°C in 10% glycerol as a cryoprotectant. For routine culture, the strain was activated (To resuscitate *Brucella* from a dormant state to active growth). on *Brucella* agar medium at 37°C in a 5% CO2 incubator for 72 h (Sacchini et al., 2019). The bacterial colonies were harvested and resuspended in sterile physiological saline, and the concentration was adjusted to 1 × 10^1^0^^ CFU/mL. The bacterial suspension was prepared fresh just before use.

### Animal grouping and infection model establishment

Female BALB/c mice (6–8 weeks old) were randomly divided into an experimental group and a control group, with 50 mice in each group. The estrous cycle of the mice was not synchronised, as the study focused on long-term dynamic changes after infection, and the magnitude of infection-induced pathological alterations was expected to far exceed cycle-related fluctuations. Mice in the experimental group were inoculated intragastrically with 1 × 10^1^0^^ CFU of *B. melitensis* 16M in a volume of 100 μL, while mice in the control group received an equal volume of sterile physiological saline (PBS) (Shang et al., 2026). The PBS blank control group was established to exclude potential interference from the intragastric administration procedure and the solvent itself on histopathological changes and bacterial detection results in the mice, while also confirming the absence of background bacterial contamination in the various organs of normal mice. Eight sampling time points were set for both groups: 1, 3 days, 1, 2, 4, 6, and 8 weeks post-infection. At each time point, five mice from each group were euthanised.

### Sample collection

At each indicated time point, mice were euthanised using the Standard of Care (SOC) euthanasia method: CO_2_ was administered at a flow rate of 3 L/min until breathing ceased (approximately 3.0–3.5 min) (Tadvalkar et al., 2020); this euthanasia method is a standard procedure commonly used in China. According to previously described methods, one gram each of the uterus, spleen, lung, and lymph nodes was collected into sterile 2 mL collection tubes pre-filled with 1 mL of PBS and zirconium oxide grinding beads. The tissues were then homogenised using a Bead Ruptor Elite bead mill homogeniser. After thorough homogenisation, the homogenates were serially diluted 10-fold and plated onto *Brucella* blood agar plates (Hensel et al., 2020). The plates were incubated at 37°C in a 5% CO_2_ incubator for 72 h, and colony-forming units (CFU) were counted. Results were expressed as the logarithm of CFU per gram of tissue (log_10_ CFU/g). Meanwhile, the spleen and uterus were weighed, and gross pathological changes were recorded.

### Histopathology and H&E staining

After euthanasia, the collected tissues (uterus, spleen, lung, and lymph nodes) were immediately fixed in 4% paraformaldehyde solution for 24–48h, then dehydrated through graded ethanol, cleared in xylene, and embedded in paraffin. Serial sections of 4μm thickness were prepared. After deparaffinisation and rehydration, sections were stained with hematoxylin and eosin (H&E): nuclei were stained with hematoxylin for 5–10min, and cytoplasm with eosin for 1–3min. Following dehydration and clearing, sections were mounted with neutral balsam. The degree of inflammatory cell infiltration, tissue structural disruption, and pathological damage in each tissue were observed under a light microscope. According to references (Hensel et al., 2019; Osman et al., 2019), semi-quantitative scoring was performed based on the pathological scoring criteria provided in Supplementary Tables 1–4. The final pathological score for each sample was the average of the scores from two observers from the pathology laboratory. Pathological evaluation often involves subjective judgment, and different observers may produce varying results due to differences in experience, perspective, or interpretation of criteria. Employing two observers allows for cross-validation, reduces bias from a single observer, and improves the accuracy and credibility of the evaluation results. When the scoring results of the two assessors were inconsistent, the following resolution protocol was adopted: the two observers first jointly re-examined the sections with substantial scoring discrepancies and attempted to reach a consensus through discussion and negotiation; if disagreement persisted after discussion, a third pathologist with a senior professional title was invited to perform an independent evaluation, and the score given by the third pathologist was taken as the final result.

### Immunohistochemistry

Paraffin sections (4μm) were deparaffinised, rehydrated, and subjected to microwave-assisted antigen retrieval using citrate buffer. Endogenous peroxidase activity was blocked by incubation with 3% H_2_O_2_ at room temperature. Non-specific binding was reduced by blocking with goat serum (ZSGB-BIO, Beijing, China) for 30min at room temperature. Subsequently, sections were incubated overnight at 4°C with a *Brucella* polyclonal rabbit antibody (Bioss, Beijing, China). After rewarming for 10min and washing, sections were incubated with a goat anti-rabbit HRP-conjugated secondary antibody (Bioss, Beijing, China) for 1h at room temperature. Color development was performed using a DAB substrate kit (ZSGB-BIO, Beijing, China), followed by counterstaining with hematoxylin, differentiation with differentiation solution for 2s, and bluing in PBS. Sections were then dehydrated, cleared, and mounted. PBS instead of the primary antibody served as a negative control to avoid false-positive results.

### Preparation of single-cell suspensions

After euthanasia, the spleen and uterus were aseptically removed, stripped of fat and connective tissue, and placed in pre-cooled PBS. Single-cell suspensions were prepared as follows. For the spleen, the organ was placed on a 70μm cell strainer and gently ground using a syringe plunger, while rinsing and filtering with DMEM (Gibco, United States) containing 10% foetal bovine serum (WILBER, Guangzhou, China) into a 15mL centrifuge tube. The cell suspension was centrifuged at 300–400 × g for 5min at 4°C, the supernatant was discarded, and red blood cells were lysed with RBC lysis buffer for 1–2min at room temperature. The reaction was stopped with PBS, followed by another centrifugation and filtration (Wu et al., 2023). The cells were resuspended for further use. Uterine immune cells were isolated according to published methods (Ji et al., 2021; Wang L. et al., 2022). After carefully removing the mesometrium, the uterus was minced into approximately 1mm^3^ fragments and placed in digestion solution containing 2mg/mL collagenase IV (Solarbio, Beijing, China), 0.4mg/mL DNase I (Solarbio, Beijing, China), and 2mg/mL hyaluronidase (Solarbio). The mixture was incubated in a shaking incubator at 37°C for 40min. The digested material was filtered through a 200-mesh sieve, centrifuged at 800 × g for 5min, and red blood cells were removed with RBC lysis buffer. After washing with PBS, a uterine single-cell suspension was obtained.

### Flow cytometry

Macrophages co-express CD11b and F4/80 (Yu et al., 2021). To assess the dynamic changes of macrophages, approximately 1 × 10^6^ viable cells were transferred to flow cytometry tubes and incubated with the following fluorochrome-conjugated antibodies: APC-Cy7-anti-CD45 (Biolegend, United States), APC-anti-CD11b (Biolegend, United States), and FITC-anti-F4/80 (Biolegend, United States), along with a live/dead cell dye (BD Biosciences, United States). The mixture was vortexed to mix and then incubated for 30 min at 4°C in the dark. After one wash with PBS, cells were resuspended. Data were acquired on a flow cytometer (BD FACS Lyric™, United States) and analyzed using FlowJo software (version 10.8.1).

### Statistical analysis

All statistical analyses were performed using GraphPad Prism 9.0 software. For bacterial load (log_10_ CFU/g) and the percentage of macrophages, at each time point the infection group was compared independently with the control group using one-way ANOVA followed by Dunnett’s multiple comparisons test (Hensel et al., 2019). For uterine and splenic weights, normality was assessed using the Shapiro–Wilk test and homogeneity of variances using Levene’s test; if both assumptions were met, an independent-samples *t-*test was used to compare the infection group with the PBS control group, otherwise the Mann–Whitney U test was applied. Histopathological scores, which were not normally distributed, were analyzed using the Kruskal–Wallis H test followed by Dunn’s test with Bonferroni correction for pairwise comparisons (Hensel et al., 2022). A *p-*value < 0.05 was considered statistically significant.

## Results

### Oral gavage with *Brucella melitensis* 16M causes multi-organ systemic infection in mice, with organ-specific bacterial colonisation kinetics

**FIGURE 1:**
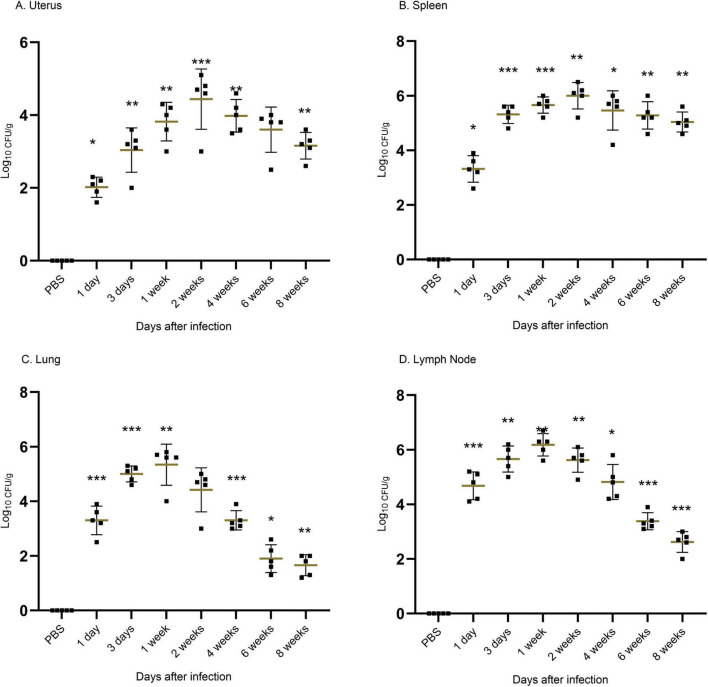
Multi-organ bacterial colonisation in mice infected with *B. melitensis 16M*. Mice were intragastrically inoculated with 1 × 10^1^0^^ CFU/100 μL of *B. melitensis* 16M. **(A)** Uterus, **(B)** spleen, **(C)** lung, and **(D)** lymph nodes were collected at 1, 3 days, 1, 2, 4, 6, and 8 weeks post-infection (*n* = 5 per group per time point). Data are presented as the mean ± standard deviation of log_10_ colony-forming units per gram of tissue (log_10_ CFU/g). A PBS negative control was included, with no viable *Brucella* recovered from mouse tissues. Comparisons against this control excluded microbial contamination and verified that all counted colonies originated from the inoculated strain. One-way ANOVA followed by Dunnett’s multiple comparison test was used to compare each infected group with the PBS control group. *p < 0.05, **p < 0.01, ***p < 0.001. In this experiment, colonies were counted after only 72 h of plate incubation, so slow-growing persisters could not be detected.

To determine whether oral inoculation with *B. melitensis 16M* induces systemic infection, bacterial loads in the uterus, spleen, lung, and mesenteric lymph nodes were quantified at 1, 3 days, 1, 2, 4, 6, and 8 weeks post-infection (Figure 1). Bacteria were detectable in all examined organs as early as 1 day after infection, indicating that *B. melitensis 16M* rapidly crosses the intestinal mucosal barrier and disseminates systemically following oral intake. In the spleen, a classical target organ, the bacterial load peaked at 2 weeks (mean ≈ 6.2 log_10_ CFU/g) and then gradually declined, but remained detectable at ≈4.6 log_10_ CFU/g at 8 weeks, suggesting that the spleen fails to completely clear the bacteria (Figure 1B). The lung and lymph nodes showed similar kinetic profiles: bacterial loads peaked at 1 week (lung: ≈5.3 log_10_ CFU/g; lymph nodes: ≈6.1 log_10_ CFU/g) and then continuously decreased; however, at 8 weeks the lymph nodes still maintained ≈2.8 log_10_ CFU/g, whereas culturable bacteria in the lung had markedly declined (Figures 1C,D). Notably, the uterus, a reproductive organ, also exhibited substantial colonisation: the bacterial load increased from 1 day post-infection (≈2.1 log_10_ CFU/g), peaked at 2 weeks (≈4.5 log_10_ CFU/g), and then steadily decreased, remaining detectable at ≈3.1 log_10_ CFU/g at 8 weeks (Figure 1A). Throughout the infection course, the bacterial load in the uterus was consistently and significantly lower than that in the spleen and lymph nodes (*p* < 0.01 or *P* < 0.001 at all-time points), suggesting that the reproductive organ may be relatively “less susceptible” to *Brucella* or may possess more active local clearance mechanisms. Bacterial loads in all organs at 2 weeks post-infection were significantly higher than those at later time points (4, 6, and 8 weeks), with highly significant differences compared with the PBS control group (one-way ANOVA with Dunnett’s multiple comparisons test, **p < 0.01 or ***p < 0.001).

### Dynamics of organ weight are not fully synchronised with bacterial load, suggesting involvement of chronic inflammation and tissue remodelling

**FIGURE 2:**
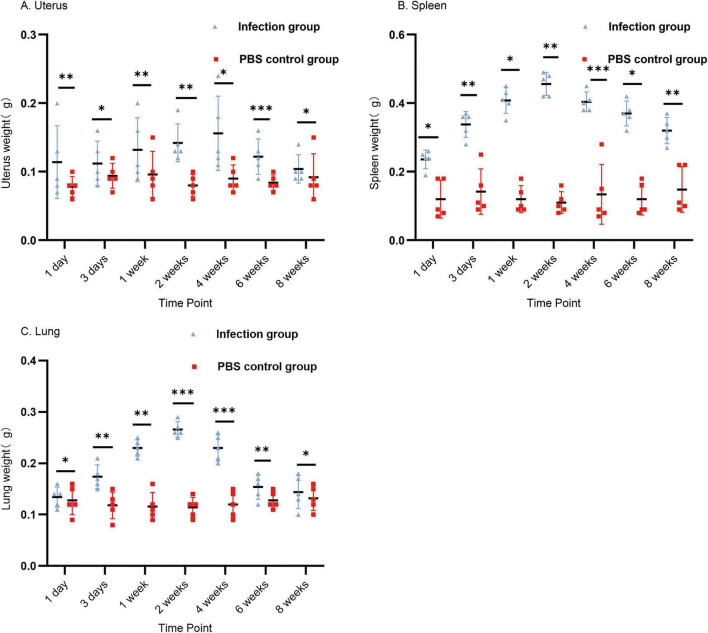
Changes in uterine and spleen weights in mice within 56 days after infection with *B. melitensis 16M*. **(A)** Uterine weight, **(B)** spleen weight and **(C)** Lung weight were measured at 1, 3 days, 1, 2, 4, 6, and 8 weeks post-infection. Mice were intragastrically inoculated with 1 × 10^1^0^^ CFU/100 μL (infected group, *n* = 5 per time point) or an equal volume of PBS (control group, *n* = 5 per time point). For uterine and splenic weights, normality was assessed using the Shapiro–Wilk test and homogeneity of variances using Levene’s test; if both assumptions were met, an independent samples *t*-test was used to compare the infected group with the PBS control group at each time point; otherwise, the Mann–Whitney U test was applied. A *p-*value < 0.05 was considered statistically significant. *p < 0.05, **p < 0.01, ***p < 0.001.

Organ weight measurements (Figure 2) revealed that uterine weight became significantly higher than that of the control group from 1 week post-infection (*p* < 0.01), continued to increase and peaked at 4 weeks (*p* < 0.05), and thereafter decreased but remained significantly elevated until 6 weeks (*p* < 0.001) (Figure 2A). Notably, the peak time of uterine weight (4 weeks) lagged behind the peak time of intrauterine bacterial load (2 weeks), and uterine weight remained high at 4–6 weeks even when the bacterial load had already begun to decline. This suggests that uterine enlargement is not only due to local bacterial colonisation but may also be related to persistent chronic inflammation, tissue oedema, and fibrotic tissue remodelling. The spleen became obviously enlarged as early as 1 day post-infection (*p* < 0.05), with splenic weight significantly higher than that of the control group from day 1, peaking at 2 weeks (*p* < 0.01), and then gradually decreasing but still remaining above the control level at 8 weeks (*p* < 0.01) (Figure 2B). Lung weight was slightly higher than that of the control group at day 1 post-infection (*p* < 0.05), then gradually increased and peaked at week 2 (*p* < 0.001), after which it slowly decreased but remained slightly above the control level at week 8 (*p* < 0.05) (Figure 2C). Similar to the spleen, the peak time of lung weight (week 2) was generally consistent with the peak time of intrapulmonary bacterial load; however, the magnitude of the increase in lung weight was relatively small, and it remained slightly higher than the control level at the late stage of infection, suggesting the possible presence of mild persistent inflammation or reparative changes in the lung.

### Histopathology and immunohistochemistry reveal organ-specific damage patterns and bacterial persistence

**FIGURE 3:**
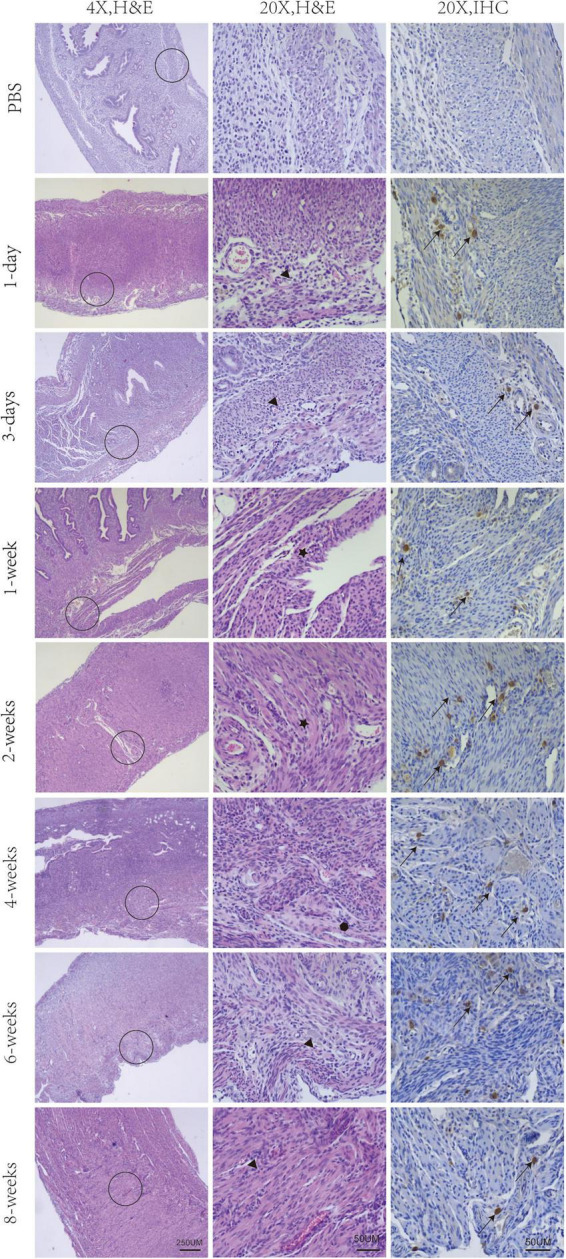
Representative histopathological and immunohistochemical images of the uterus from mice infected with *B. melitensis 16M*. Uterine tissues were collected from mice intragastrically inoculated with 1 × 10^1^0^^ CFU of *B. melitensis* 16M at 1, 3 days, 1, 2, 4, 6, and 8 weeks post-infection, as well as from a healthy control group receiving PBS. The black circles in the left column indicate the regions shown at higher magnification in the middle column. *B. melitensis* infection caused progressive inflammatory cell infiltration (indicated by ▲), tissue edema (indicated by ★) and granulation tissue (indicated by 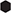). Immunohistochemistry revealed Brucella antigen in the cytoplasm of macrophages within the inflammatory areas (indicated by arrows). Magnification: left column, 4 × (H&E staining, scale bar = 250 μm); middle column, 20 × (H&E staining, scale bar = 50 μm); right column, 20 × (DAB chromogenic immunohistochemistry, scale bar = 50 μm).

**FIGURE 4:**
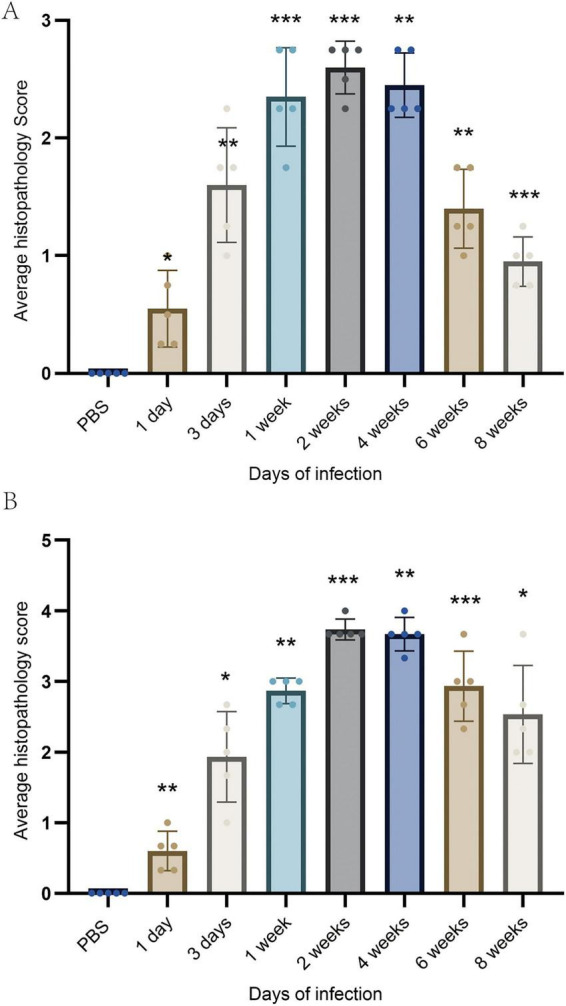
Dynamic histopathological scores of the uterus **(A)** and spleen **(B)** from mice infected with *B. melitensis 16M*. Histopathological scores of the uterus **(A)** and spleen **(B)** at different time points after intragastric inoculation with 1 × 10^1^0^^ CFU of *B. melitensis* 16M. Scoring was performed according to the criteria provided in the Supplementary Materials. Data are presented as the mean ± SD. Comparisons between each infected group and the PBS control group were performed using the Kruskal–Wallis H test followed by Dunn’s test with Bonferroni correction for pairwise comparisons. *p < 0.05, **p < 0.01, ***p < 0.001.

Based on the bacterial colonisation kinetics results described above (Figure 1), we further evaluated tissue damage and bacterial distribution in various organs by H&E staining and anti-*Brucella* immunohistochemistry (IHC). The results are as follows:

Uterus (Figures 3, 4A): No obvious pathological changes were observed in the uterine tissue of the PBS control group (score = 0). On days 1 and 3 post-infection, mild endometrial edema and a few scattered inflammatory cells (mainly macrophages and neutrophils) appeared in the uterus. The histopathological scores were 0.55 ± 0.32 and 1.6 ± 0.48, respectively, both significantly higher than those of the control group (*p* < 0.05 and *P* < 0.01). IHC revealed that *Brucella* antigens could be detected in the serosal layer as early as day 1 post-infection; subsequently, the antigens spread to the myometrium and the endometrial lamina propria, indicating the ability of the bacteria to traverse the uterine wall. From the first week post-infection onwards, the pathological changes worsened. The score increased to 2.35 ± 0.41 (*p* < 0.01), with mild endometrial edema and scattered inflammatory cells observed microscopically. The pathological alterations peaked during weeks 2–4 post-infection, characterised by glandular atrophy, marked stromal congestion and edema, as well as diffuse infiltration of lymphocytes, macrophages, and a few neutrophils. The histopathological score reached its maximum at week 2 (2.60 ± 0.22, *p* < 0.01). At weeks 4, 6, and 8, the scores decreased to 2.45 ± 0.27, 1.40 ± 0.33, and 0.95 ± 0.20, respectively (all *p* < 0.05). By week 8, the lesions had markedly subsided, although focal lymphoplasmacytic infiltration still remained in the stroma, and the IHC signal, albeit weaker, remained detectable.

**FIGURE 5:**
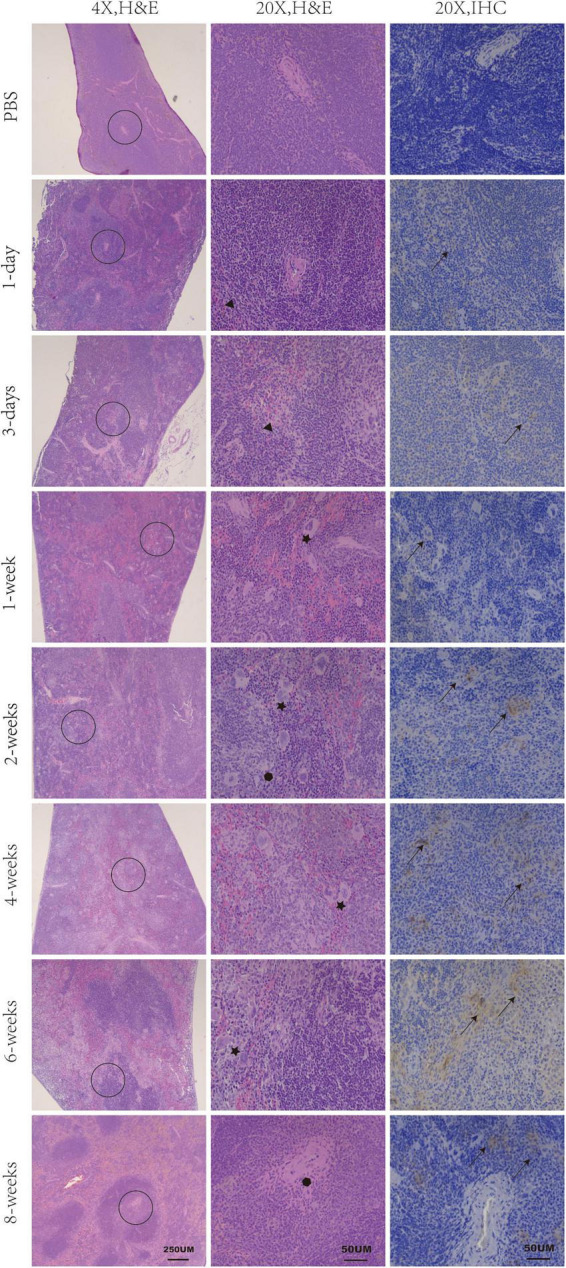
Representative histopathological and immunohistochemical images of the spleen from mice infected with *B. melitensis 16M*. Spleen tissues were collected from mice intragastrically inoculated with 1 × 10^1^0^^ CFU of *B. melitensis* 16M at 1, 3 days, 1, 2, 4, 6, and 8 weeks post-infection, as well as from a healthy control group receiving PBS. The black circles in the left column indicate the regions shown at higher magnification in the middle column. *B. melitensis* infection caused progressive, inflammatory cell infiltration (indicated by ▲), macrophage infiltration (indicated by ★) and granulation tissue (indicated by 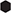). Leading to disruption of the normal splenic architecture. Immunohistochemistry revealed Brucella antigen in the cytoplasm of macrophages within the microgranulomas and inflammatory areas (indicated by arrows).

Spleen (Figures 4B, 5): The spleen of the PBS control group showed a normal structure with no pathological changes (score = 0). On days 1 and 3 post-infection, mild congestion and scattered inflammatory cell infiltration appeared in the spleen. The histopathological scores were 0.60 ± 0.28 and 1.93 ± 0.64, respectively, both significantly higher than those of the control group (*p* < 0.01 and *p* < 0.05). IHC showed that *Brucella* antigens could be detected in the marginal zone of the white pulp as early as day 1 post-infection. From the first week post-infection onwards, the pathological changes markedly worsened: diffuse infiltration of macrophages and neutrophils and extensive congestion were observed in the red pulp, and the score increased to 2.86 ± 0.18 (*p* < 0.01). By week 2, the spleen was significantly enlarged, with extensive formation of microgranulomas composed of aggregated epithelioid macrophages, destruction of the normal architecture, and blurring of the white pulp/red pulp boundary. IHC-positive areas were widely distributed throughout the red pulp, consistent with the bacterial load distribution. The histopathological score peaked at week 2 (3.73 ± 0.14, *P* < 0.001); scores decreased in the following weeks (week 4: 3.66 ± 0.14, *p* < 0.01; week 6: 2.93 ± 0.49, *P* < 0.001; week 8: 2.53 ± 0.69, *p* < 0.05), although the most typical microscopic changes were observed at week 2. At week 8, widening of the red pulp cords, disorganisation of lymphoid follicles, and residual microgranulomas were still observable. The IHC-positive signal, albeit reduced, persisted, suggesting that splenic injury was progressively aggravated and incompletely repaired, consistent with bacterial persistence and chronic inflammation.

**FIGURE 6:**
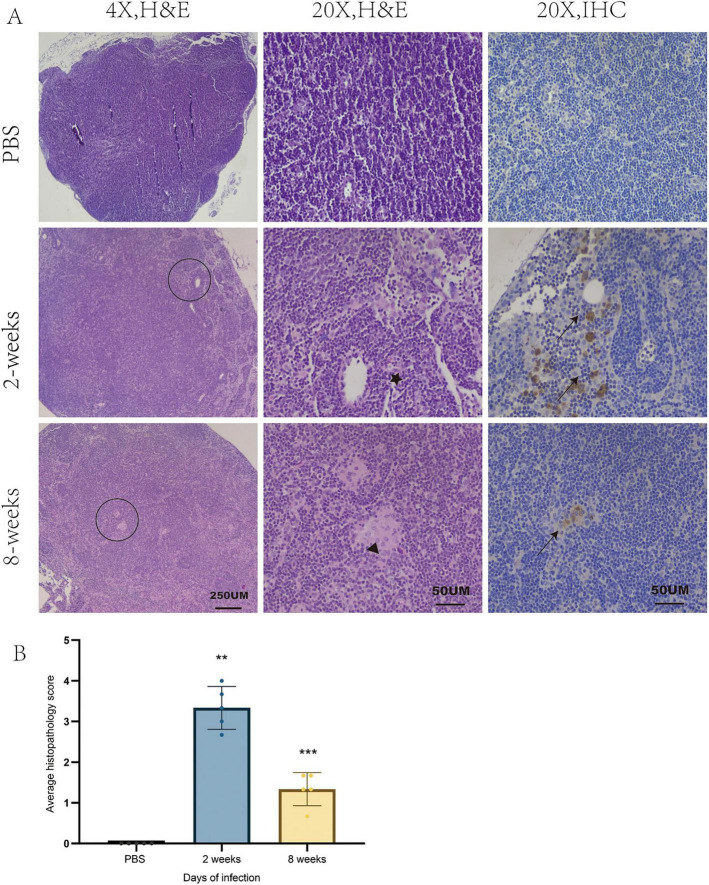
Representative histopathological and immunohistochemical images of lymph nodes from mice infected with *B. melitensis 16M*, and dynamic histopathological scores. **(A)** Representative histopathological and immunohistochemical images of lymph nodes from mice intragastrically inoculated with 1 × 10^1^0^^ CFU of *B. melitensis* 16M, including a healthy control group receiving PBS (PBS), and infected groups at 2 and 8 weeks post-infection. The black circles in the left column indicate the regions shown at higher magnification in the middle column. At 14 days post-infection (2 weeks), *B. melitensis* infection caused extensive inflammatory cell infiltration (indicated by ▲) and disruption of the normal lymph node structure (indicated by ★). At 56 days post-infection (8 weeks), the inflammatory response was markedly reduced, with residual microgranulomas and mild inflammatory infiltration still observable. Immunohistochemistry revealed Brucella antigen in the cytoplasm of macrophages within the microgranulomas (indicated by arrows); abundant positive signals were present during the acute phase, while signals decreased but persisted during the chronic phase. **(B)** Dynamic histopathological scores of lymph nodes at different time points after intragastric infection. Scoring was based on the degree of inflammatory infiltration and tissue damage in H&E-stained sections. Data are presented as the mean ± SD. Comparisons among multiple groups were performed using the Kruskal-Wallis H test, followed by Dunn’s *post hoc* test with Bonferroni correction. **p < 0.01, ***p < 0.001 compared with the PBS control group.

Lymph nodes (Figures 6A,B): The lymph nodes of the PBS control group showed a normal structure, with a histopathological score of 0. At week 2 post-infection, the lymph nodes were significantly enlarged, with follicular hyperplasia in the cortex, marked congestion in the medulla, and abundant macrophage infiltration and microgranuloma formation in the paracortex and medulla. IHC revealed abundant bacterial aggregation in the lymph nodes.

The histopathological score was significantly elevated at week 2 (3.33 ± 0.52), which was highly statistically significant compared with the PBS control group (*p* < 0.01). By week 8 post-infection, the lesions had partially resolved: the structure of lymphoid follicles tended to normalise, but scattered microgranulomas and multinucleated giant cells still remained in the medulla. IHC still showed weak residual positive signals in the lymph nodes, suggesting that the bacteria may persist as persisters. The histopathological score at week 8 decreased to 1.33 ± 0.40, which remained significantly higher than that of the PBS control group (*p* < 0.001) and was significantly lower than the week-2 score.

**FIGURE 7:**
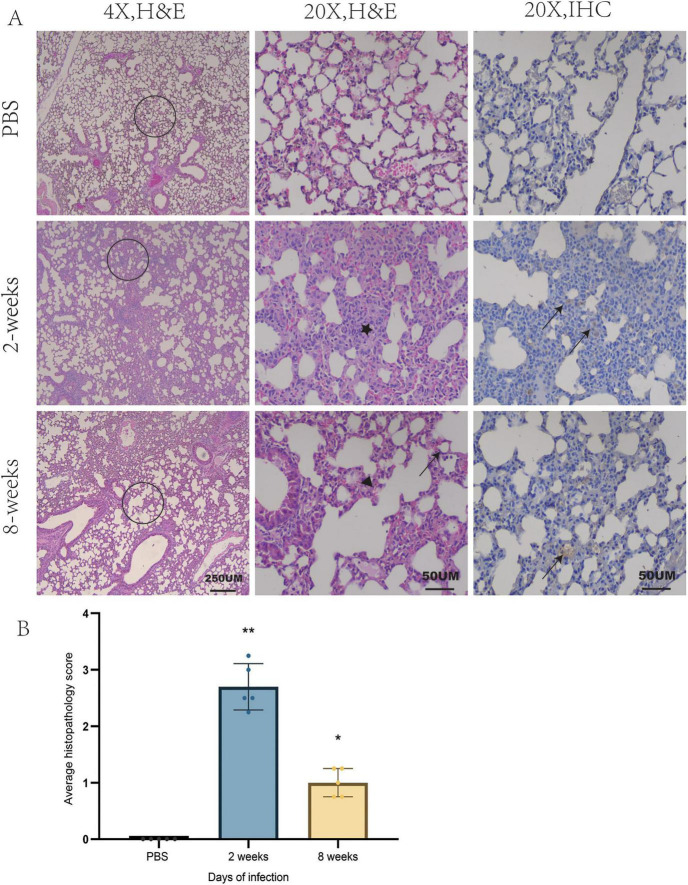
Representative histopathological and immunohistochemical images of lung tissue from mice infected with *B. melitensis 16M*, and dynamic histopathological scores. **(A)** Representative histopathological and immunohistochemical images of lung tissue from mice intragastrically inoculated with 1 × 10^1^0^^ CFU of *B. melitensis* 16M, including a healthy control group receiving PBS (PBS), and infected groups at 2 and 8 weeks post-infection. The black circles in the left column indicate the regions shown at higher magnification in the middle column. The healthy control group showed intact alveolar structures, clear alveolar spaces, and no inflammatory cell infiltration (★ indicates thickened alveolar walls, ▲ indicates thickened alveolar walls gradually recovered). At 2 weeks post-infection, *B. melitensis* infection caused marked interstitial inflammatory cell infiltration, alveolar wall thickening, and pulmonary consolidation, with disruption of normal alveolar architecture. At 8 weeks post-infection, the inflammatory infiltration was markedly reduced; only mild alveolar wall thickening was observed, with no obvious interstitial inflammatory cell infiltration. Immunohistochemistry revealed Brucella antigen in the cytoplasm of macrophages within the inflamed interstitial areas (indicated by arrows). **(B)** Dynamic histopathological scores of lung tissue at different time points after intragastric infection. Scoring was based on the degree of inflammatory infiltration and tissue damage in H&E-stained sections. Data are presented as the mean ± SD. Comparisons among multiple groups were performed using the Kruskal-Wallis H test, followed by Dunn’s *post hoc* test with Bonferroni correction. *p < 0.05, **p < 0.01 compared with the PBS control group.

Lung (Figures 7A,B): The lung tissue of the PBS control group showed a normal structure, with clear alveolar septa and no inflammatory cell infiltration; IHC showed that the bacteria were localised in the pulmonary interstitium. the histopathological score was 0. At week 2 post-infection, thickening of the alveolar septa, interstitial inflammatory cell infiltration, and small focal necrosis were observed. IHC showed bacteria localised in the cytoplasm of interstitial macrophages. The histopathological score was significantly elevated at week 2 (2.70 ± 0.41), which was statistically significant compared with the PBS control group (*p* < 0.01). By week 8 post-infection, the inflammation had markedly subsided, the alveolar structure was largely restored, the IHC signal was significantly reduced, and only occasional weakly positive macrophages were seen. The score at week 8 decreased to 1.00 ± 0.25, which, although still higher than that of the PBS control group (*p* < 0.05), was significantly lower than the week-2 score, indicating repair of pathological damage. Of note, tertiary lymphoid structures (TLS) were observed in the lung tissue of 1/5 mice, suggesting the induction of a local adaptive immune response. This stands in contrast to the relatively effective bacterial clearance in the lung compared with other organs.

### The proportion of F4/80^+^CD11b^+^ macrophages in the uterus and spleen shows a dynamic pattern of initial increase followed by suppression

**FIGURE 8:**
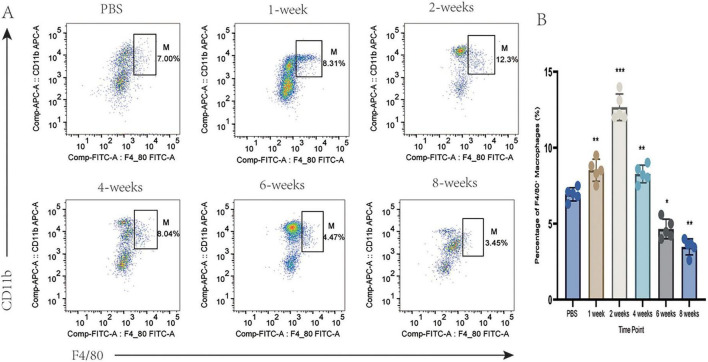
Dynamic changes of F4/80^+^CD11b^+^ macrophages in the uterus of mice infected with *B. melitensis 16M*. **(A)** Representative flow cytometry scatter plots and gating strategy for F4/80^+^CD11b^+^ macrophages in the uterus of mice from the PBS-treated healthy control group and infected groups at 1, 2, 4, 6, and 8 weeks post-infection. The percentage of F4/80^+^CD11b^+^ macrophages is indicated for each group. **(B)** Quantitative analysis of the percentage of F4/80^+^CD11b^+^ macrophages. Data are presented as the mean ± standard error of the mean (SEM) (*n* = 5 mice per group). One-way ANOVA followed by Dunnett’s multiple comparison test was used to compare each infected group with the PBS control group. A *p*-value < 0.05 was considered statistically significant. **p < 0.01, ***p < 0.001.

**FIGURE 9:**
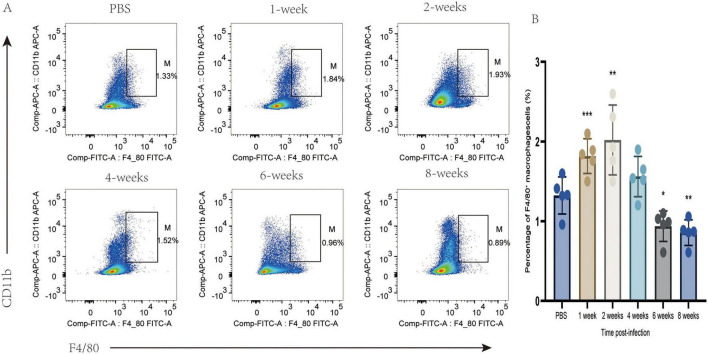
Dynamic changes of F4/80^+^CD11b^+^ macrophages in the spleen of mice infected with *B. melitensis 16M*. **(A)** Representative flow cytometry scatter plots and gating strategy for F4/80^+^CD11b^+^ macrophages in the spleen of mice from the PBS-treated healthy control group and infected groups at 1, 2, 4, 6, and 8 weeks post-infection. The percentage of F4/80^+^CD11b^+^ macrophages is indicated for each group. **(B)** Quantitative analysis of the percentage of F4/80^+^CD11b^+^ macrophages. Data are presented as the mean ± standard error of the mean (SEM) (*n* = 5 mice per group). One-way ANOVA followed by Dunnett’s multiple comparison test was used to compare each infected group with the PBS control group. A *p*-value < 0.05 was considered statistically significant. **p < 0.01, ***p < 0.001; ns, not significant.

Flow cytometry was used to determine the proportion of F4/80^+^CD11b^+^ macrophages among CD45^+^ leukocytes in the uterus and spleen (gating strategy shown in Supplementary Figures 1, 2). The results (Figures 8, 9) are as follows.

Uterus (Figure 8): Compared with the PBS healthy control group, the proportion of macrophages began to increase at 1 week post-infection, peaked at 2 weeks (*p* < 0.001), remained higher than the control group at 4 weeks but with a significant decrease (*p* < 0.01), fell below the control group at 6 weeks (*p* < 0.05), and further decreased at 8 weeks (*p* < 0.01). This dynamic is partially synchronised with the uterine bacterial load (peak at 2 weeks), but the macrophage proportion rapidly fell below baseline after the bacterial load declined, suggesting that *Brucella* may suppress local macrophage recruitment or induce apoptosis/depletion during the late phase of infection (Dornand et al., 2002; Giambartolomei and Delpino, 2019) Spleen (Figure 9):

A similar trend was observed: the proportion of macrophages increased at 1 week post-infection, peaked at 2 weeks (*p* < 0.01), showed a declining trend at 4 weeks without statistical significance (ns), was significantly lower than the control group at 6 weeks (*p* < 0.05), and further decreased at 8 weeks (*p* < 0.01). The peak time of splenic macrophage proportion (2 weeks) coincided exactly with the peak splenic bacterial load and peak splenomegaly, suggesting that macrophages are massively mobilised to the spleen during the acute phase to control the bacteria, but immune exhaustion occurs after prolonged infection.

Taken together, the combined histopathological, IHC, and flow cytometry results demonstrate that oral gavage with *B. melitensis 16M* leads to rapid multi-organ dissemination, with peaks of bacterial colonisation and pathological damage occurring around 2 weeks post-infection, whereas the peak of macrophage proportion occurs between 1 and 4 weeks. The uterus, spleen, and lymph nodes show partial tissue repair but persistent bacteria during the chronic phase (8 weeks), whereas the lung undergoes better repair and effectively clears the bacteria. These findings provide a systematic experimental basis for understanding the systemic dissemination pattern, organ tropism differences, and local macrophage dynamics following oral infection with *Brucella*.

## Discussion

This study systematically describes the bacterial colonisation kinetics, histopathological evolution, immunohistochemical features, and dynamic changes of local F4/80^+^CD11b^+^ macrophages in multiple organs (uterus, spleen, lung, and lymph nodes) of mice over an 8-week period following intragastric infection with *B. melitensis 16M*. The main findings are as follows: (1) *Brucella* rapidly disseminates systemically after oral intake, with bacteria detectable in all examined organs at the early stage of infection; (2) the time to peak bacterial load and pathological damage varies among organs, with the spleen, uterus, and lymph nodes peaking at 2 weeks and the lung at 1 week; (3) in the chronic phase (8 weeks), bacteria persist with partial pathological repair in the uterus, spleen, and lymph nodes, whereas the lung eventually exhibits a low bacterial burden; (4) the proportion of F4/80^+^CD11b^+^ macrophages among CD45^+^ cells in the uterus and spleen increases significantly from 1 to 4 weeks post-infection, peaks at 2 weeks, and then decreases to levels below those of the PBS control group by 8 weeks.

Compared with the commonly used intraperitoneal injection or aerosol infection routes in previous studies (Stranahan et al., 2019; Ferrero et al., 2020; Hensel et al., 2022), intragastric administration more closely mimics the natural process of human infection with *Brucella* through contaminated food or water. In the present study, bacteria were detectable in all organs as early as 1 day post-infection, indicating that *Brucella* rapidly crosses the gastrointestinal barrier and disseminates systemically (Ferrero et al., 2020). This result is consistent with reports that *Brucella* can undergo transcellular transport mediated by intestinal epithelial cells and dendritic cells, leading to metastatic infection (Ferrero et al., 2012; Avila-Calderón et al., 2020). Notably, although the bacterial load in the lung rapidly decreased after peaking at 1 week, it remained detectable until 8 weeks, suggesting that *Brucella* has the ability to establish long-term latency in organs, which is consistent with the clinical characteristics of human brucellosis that tends to become chronic (Rajashekara et al., 2006; Dean et al., 2012).

Most previous mouse models of oral infection used sodium bicarbonate to neutralise gastric acid in order to increase the success rate of infection (Pasquali et al., 2003). In this study, we did not use this pretreatment; instead, the bacterial suspension was directly administered by intragastric gavage into the normal gastric acid environment. If sodium bicarbonate pretreatment had been used, it might have affected the measured indicators as follows: (1) Bacterial burden: neutralising gastric acid would increase bacterial survival in the stomach, and it is expected that the peak bacterial load in various organs would be higher and the time to peak would be advanced. (2) Histopathological damage: with increased bacterial load after neutralisation, more pronounced lesions such as uterine glandular atrophy and splenic microgranulomas would be expected, with higher peak pathological scores and delayed recovery; it might also induce an excessive acute immune response due to massive and rapid bacterial entry into the intestine. (3) Macrophage dynamics: with increased bacterial load after neutralisation, the peak proportion of F4/80^+^CD11b^+^ macrophages would likely be higher and the duration of high expression prolonged. The non-neutralisation model more closely resembles natural oral infection in humans (gastric acid barrier); the neutralisation model is more appropriate for high-dose exposure or gastric acid deficiency scenarios.

Compared with previous studies, our study has both similarities and novel extensions. Paixão et al. (2009) orally infected mice with *B. melitensis 16M* and found that bacteria could be consistently isolated from mesenteric lymph nodes (MLN), spleen, and liver from 3 to 7 days post-infection, and persisted in the intestine up to 21 days, but no inflammatory lesions were observed in the ileum or colon. Our results are partially consistent with that study: we also observed that bacteria could be detected in the spleen and lymph nodes early after infection, and the bacterial load in the spleen peaked at week 2. However, our study further found substantial bacterial colonisation and histopathological damage in reproductive organs such as the uterus, which were not examined in that study (Paixão et al., 2009). Using a mouse model of oral infection, the virulence factors required for *Brucella* to establish oral infection were systematically evaluated. The results demonstrated that urease (the ureABC gene deletion mutant showed significantly reduced colonisation in the spleen), the type IV secretion system (T4SS; the virB2 deletion mutant exhibited significantly reduced colonisation in the mesenteric lymph nodes), and the lipopolysaccharide (LPS) O-antigen (the pmm deletion mutant displayed significantly reduced colonisation in the mesenteric lymph nodes) are all essential virulence factors for *B. melitensis* to successfully establish oral infection, traverse the digestive tract barrier, and achieve systemic dissemination (Paixão et al., 2009). Moreover, while that study reported no intestinal inflammation, we observed significant inflammatory responses in organs such as the uterus and spleen, suggesting heterogeneity in the responses of different organs to *Brucella* infection. Izadjoo et al. (2008) compared the oral infection process of the attenuated strain WR201 with the virulent strain 16M in male BALB/c mice and found that 16M infection caused significant inflammation in reproductive organs such as the testis and epididymis, whereas the attenuated strain did not. Using female mice, our study similarly found significant pathological damage and bacterial colonisation in a reproductive organ (the uterus) after 16M infection, with the peak of damage (weeks 2–4) coinciding with that in the spleen and lymph nodes. Comparing the results of the two studies, it can be inferred that *B. melitensis* is capable of invading reproductive organs in both male and female mice, and the pathological changes in the female uterus and the inflammatory responses in the male testis/epididymis exhibit similar temporal dynamics. Delpino et al. (2007) identified choline-glycine hydrolase (CGH) in *Brucella*, demonstrating that it confers resistance to bile salts, and confirmed by oral infection experiments that a CGH deletion mutant had significantly reduced colonisation capacity in the mouse spleen, indicating that CGH is an important virulence factor for oral infection by *Brucella*. That study provided a molecular explanation for how *Brucella* adapts to the digestive tract environment. Combining their findings with our results, we speculate that after oral infection, *Brucella* may first resist the bactericidal action of bile salts through factors such as CGH, then traverse the intestinal epithelial barrier via M cells, and subsequently disseminate to various organs throughout the body via the lymphatic and blood circulation.

The present study found that the bacterial colonisation pattern in the uterus was distinctly different from that in immune organs. The uterine bacterial load peaked at 2 weeks post-infection, whereas the uterine weight peaked at 4 weeks and the histopathological score peaked at 2 weeks. The later peak of uterine weight compared with the bacterial load suggests that uterine enlargement depends not only on the local bacterial burden but may also be related to persistent inflammation and tissue remodelling induced by the infection (Klimaszyk et al., 2023). Immunohistochemical results showed a tendency of *Brucella* antigen to gradually break through from the serosal layer toward the endometrial layer in the uterus, which may represent an important strategy for the pathogen to establish deep infection and evade host clearance. This experiment confirmed that, in non-pregnant female mice, oral infection with *B. melitensis 16M* leads to uterine colonisation and inflammation. Further analysis integrating existing literature on pregnant mouse models indicates that after *Brucella* infection in pregnant mice, the bacteria can heavily colonise placental trophoblast cells, inducing necrotising placentitis and resulting in abortion (Tobias et al., 1993). Compared with the non-pregnant uterus, bacterial colonisation in the placenta of pregnant mice is more intense and the damage is more severe, which is associated with the progesterone environment and the susceptibility of trophoblast cells (Elizalde-Bielsa et al., 2024). The bacterial colonisation and macrophage infiltration in the non-pregnant uterus observed in this study may provide a latent precursor for severe placental infection after pregnancy.

As the primary target organs of *Brucella*, the spleen and lymph nodes allow the bacterium to establish long-term colonisation within the replicative compartment of macrophages, thereby evading the host’s bactericidal mechanisms (Verdiguel-Fernández et al., 2020; Qin et al., 2024). The immunohistochemical results of this study showed that *Brucella* antigens could still be detected in the cytoplasm of macrophages in lymph nodes during the chronic phase, further confirming this characteristic. Unlike the spleen and lymph nodes, the lungs exhibited marked resolution of inflammation and a significant reduction in bacterial signals during the chronic phase. This difference may be attributed to the high phagocytic activity of resident alveolar macrophages and a more effective adaptive immune response (Dadar et al., 2025). Furthermore, following intragastric inoculation, bacteria first reach the liver and spleen via the portal vein; the lungs are not a primary colonisation organ, and the transient bacterial presence there may be more readily cleared (Machelart et al., 2017).

In the present study, we comparatively analyzed the bacterial loads (CFU/g) in multiple organs and the results of anti-Brucella immunohistochemistry (IHC) staining at different time points post-infection. The two detection methods exhibited an overall consistent trend, while inconsistent findings were observed in certain organs and at individual time points. For the spleen: The bacterial load peaked at week 2 post-infection, accompanied by extensive positive staining in IHC sections. By week 8 post-infection, the bacterial load decreased, together with a concurrent reduction in the intensity and distribution of IHC-positive signals, indicating a positive correlation between the IHC staining level and viable bacterial counts in the spleen. For the uterus: IHC-positive signals were detectable as early as day 1 post-infection, and bacterial isolation confirmed low-level Brucella colonisation. Both indicators peaked at week 2, with bacterial antigens spreading to the myometrium and endometrial lamina propria shown by IHC. At the late infection stage (weeks 6–8), although viable bacterial loads declined to a low level, weak positive signals were still observed in the uterine interstitium, which was possibly attributed to the persistence of a small number of persistent bacteria. For the lung: Pulmonary bacterial load also reached its maximum at week 2 post-infection, yet only scattered positive signals rather than extensive staining as seen in the spleen were found within the pulmonary interstitium via IHC. This could be explained by the oral gavage infection route: bacteria disseminate primarily via the digestive tract, resulting in severe injury to digestive organs and relatively mild pathological damage to the respiratory system. Similarly, the lymph nodes displayed high bacterial burdens at week 2 followed by obvious declines at week 8, which was well consistent with the corresponding IHC staining outcomes.

Flow cytometry results showed that during the 1–4 week period post-infection, the proportion of F4/80^+^CD11b^+^ macrophages among CD45^+^ cells in the uterus and spleen was significantly increased, with both organs peaking at 2 weeks post-infection. Although the timing of the peak macrophage proportion was the same in the uterus and spleen, the underlying mechanisms may be tissue-specific: the increase in the splenic macrophage proportion is likely driven directly by local bacterial proliferation (Machelart et al., 2017), whereas the increase in the uterine macrophage proportion is more likely related to monocyte recruitment induced by chemokines and inflammatory mediators released early during infection (Wang et al., 2020; Fattori et al., 2024). By 8 weeks post-infection, the macrophage proportion in both organs had returned to baseline levels, consistent with the resolution of chronic inflammation and partial repair of histopathological damage (Oishi and Manabe, 2018). However, despite the decrease in the proportion of macrophages among CD45^+^ cells, *Brucella* continued to colonise both organs, suggesting that the bacterium successfully establishes an immune-evasive state. The mechanisms may involve immunomodulatory effects such as inhibition of macrophage apoptosis and induction of M2-type macrophage polarisation (Wang et al., 2017; Wang Y. et al., 2022; Zhao et al., 2023).

This study systematically describes the dynamic pathological and immunological changes in multiple organs of mice after oral infection with *Brucella*, filling a data gap for the oral infection model in this field. The results provide important experimental evidence for understanding the chronicity of brucellosis and the differences in organ tropism. Moreover, the multi-time-point, multi-parameter evaluation system established in this study can serve as a reference model for evaluating the efficacy of future anti-*Brucella* drugs, vaccines, or immunomodulators.

The following limitations should be acknowledged. First, cytokine profiles and macrophage polarisation phenotypes (M1/M2) were not examined, limiting in-depth exploration of the regulatory mechanisms of the immune microenvironment. Second, only BALB/c mice were used, and the response in different genetic backgrounds was not compared. Third, to ensure stable infection in all mice and facilitate dynamic observation at multiple time points, a relatively high dose (1 × 10^1^0^^ CFU) was used for intragastric inoculation, which may differ from the low-dose exposure that occurs in natural infection. Future dose titration studies could evaluate the pathological progression at lower doses. Fourth, the estrous cycle of female mice was not synchronised. Although the inflammatory and immune status of the uterus is influenced by hormones, the severity of uterine lesions (e.g., diffuse inflammatory cell infiltration, glandular atrophy) and the marked changes in macrophage proportion (*p* < 0.001) at 2 and 4 weeks post-infection exceed the physiological fluctuations during the normal estrous cycle. Therefore, we believe that the lack of estrous cycle synchronisation does not affect the main conclusions of this study.

## Conclusion

Oral *B. melitensis* infection Leads to persistent bacterial colonisation and dynamic histopathology in reproductive and immune organs of female mice. In this study, intragastric inoculation with *B. melitensis 16M* led to systemic multi-organ infection in mice. The peak of pathology and bacterial colonisation occurred at 2 weeks post-infection, while the peak of macrophage proportion occurred between 1 and 4 weeks. In reproductive (uterus) and immune organs (spleen and lymph nodes), partial tissue repair was observed during the chronic phase, but bacteria persisted; in contrast, the lung showed good repair of tissue damage. This study systematically describes the dynamic pathological and immunological changes in multiple organs of female mice after oral infection, providing an important mouse model basis for studies on the pathogenesis of brucellosis and vaccine evaluation, with a particular emphasis on bacterial persistence in both reproductive and immune tissues.

## Data Availability

The original contributions presented in this study are included in the article/Supplementary material, further inquiries can be directed to the corresponding authors.
