# Supplementary material for: Oral Brucella melitensis infection leads to persistent bacterial colonisation and dynamic histopathology in reproductive and immune organs of female mice
Source: Front Microbiol. 2026 Jul 6;17:1871530. doi: 10.3389/fmicb.2026.1871530 (PMC13381521; doi:10.3389/fmicb.2026.1871530)
Supplement: Supplementary file 1 [file Data_Sheet_1.docx]

**Supplementary figure 1**


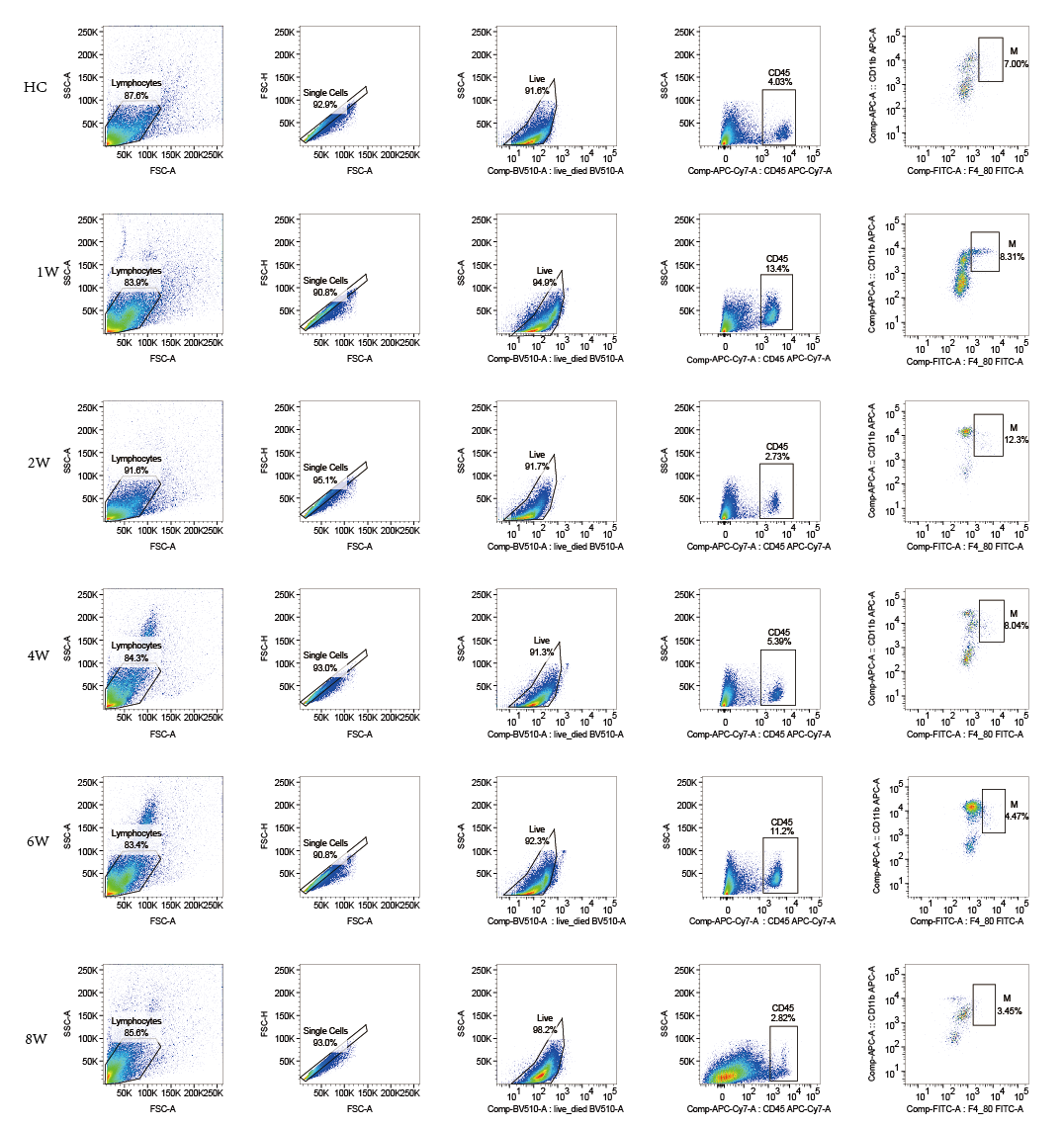


**Figure S1.** Representative flow cytometry gating strategy for macrophage identification in uterine tissue. Cells were first gated based on forward scatter (FSC) and side scatter (SSC) to exclude debris and cell doublets. Subsequently, single-cell gating (FSC-A/FSC-H), live cell gating (negative for live/dead stain), and CD45⁺ leukocyte gating were performed sequentially. Finally, CD11b⁺F4/80⁺ macrophage populations were identified within the CD45⁺ cell population. Percentages shown indicate the proportion of cells retained at each gating step. Representative gating plots for macrophages in uterine tissue from the PBS control group (HC) and mice at 1, 2, 4, 6, and 8 weeks post *Brucella* infection are shown on the right.

**Supplementary figure 2**


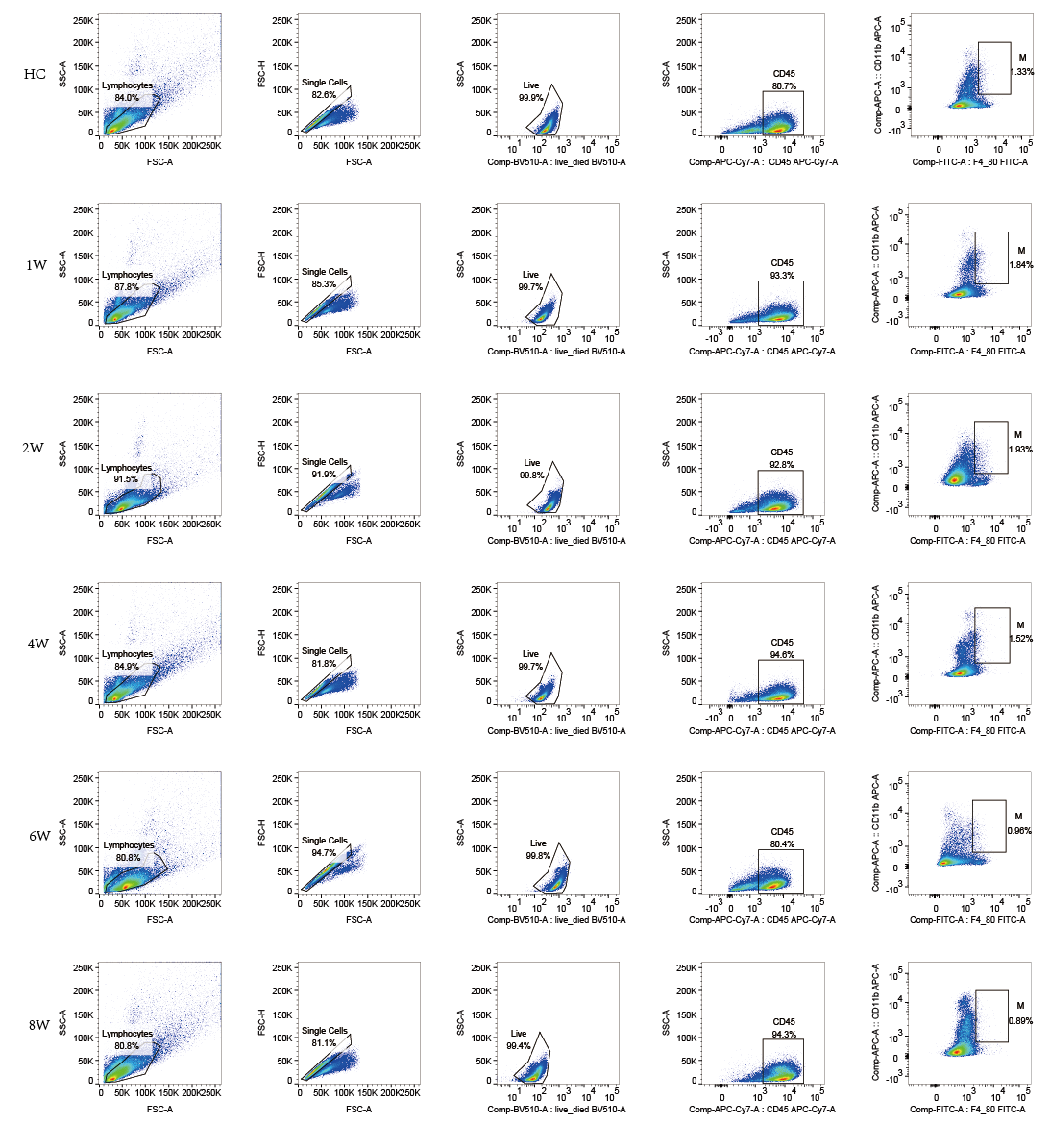


**Figure S2.** Representative flow cytometry gating strategy for macrophage identification in spleen tissue. Cells were first gated based on forward scatter (FSC) and side scatter (SSC) to exclude debris and cell doublets. Subsequently, single-cell gating (FSC-A/FSC-H), live cell gating (negative for live/dead stain), and CD45⁺ leukocyte gating were performed sequentially. Finally, CD11b⁺F4/80⁺ macrophage populations were identified within the CD45⁺ cell population. Percentages shown indicate the proportion of cells retained at each gating step. Representative gating plots for macrophages in spleen tissue from the PBS control group (HC) and mice at 1, 2, 4, 6, and 8 weeks post *Brucella* infection are shown on the right.
