# Supplementary material for: Oral Brucella melitensis infection leads to persistent bacterial colonisation and dynamic histopathology in reproductive and immune organs of female mice
Source: Front Microbiol. 2026 Jul 6;17:1871530. doi: 10.3389/fmicb.2026.1871530 (PMC13381521; doi:10.3389/fmicb.2026.1871530)
Supplement: Supplementary file 2 [file Table_1.docx]

**Table 1. Histopathological scoring criteria for spleen.**

| **Spleen** | Score | Description |
| --- | --- | --- |
| **Histiocytic inflammation** | 0 | None |
|  | 1 | Minimal—One focus per 4x objective |
|  | 2 | Mild—Two to four foci per 4x objective |
|  | 3 | Moderate—Five to 10 foci per 4x objective |
|  | 4 | Marked—>10 per 4x objective |
| **Neutrophilic accumulation** | 0 | None |
|  | 1 | Minimal—few cells identified |
|  | 2 | Mild—multiple small foci <10 cells |
|  | 3 | Moderate—1-2 foci of >10 cells |
|  | 4 | Marked—Multiple foci of >10 cells |
| **Necrosis** | 0 | None |
|  | 1 | Minimal—one focus per 10x objective |
|  | 2 | Mild—Two to four foci per 10x objective |
|  | 3 | Moderate—Five to ten foci per 10x objective |
|  | 4 | Marked—>10 foci per 10x objective |

**Table 2. Histopathological scoring criteria for Uterus.**

| **Uterus** | Score | Description |
| --- | --- | --- |
| **Myometrial inflammation** | 0 | None |
|  | 1 | Minimal—One focus per 4x objective |
|  | 2 | Mild—Two to four foci per 4x objective |
|  | 3 | Moderate—Five to 10 foci per 4x objective |
|  | 4 | Marked—>10 per 4x objective |
| **Endometrial neutrophilic inflammation** | 0 | None |
|  | 1 | Minimal—few cells identified |
|  | 2 | Mild—multiple small foci <10 cells |
|  | 3 | Moderate—1-2 foci of >10 cells |
|  | 4 | Marked—Multiple foci of >10 cells |
| **Edema** | 0 | None |
|  | 1 | Present |

**Table 3. Histopathological scoring criteria for Lymph Node**

| **Lymph Node** | Score | Description |
| --- | --- | --- |
| **Follicular hyperplasia** | 0 | None |
|  | 1 | Minimal — Slight increase in follicle size/number |
|  | 2 | Mild — Moderate increase in follicle size/number |
|  | 3 | Moderate — Marked increase in follicle size/number |
|  | 4 | Marked — Diffuse hyperplasia, architecture distortion |
| **Histiocytic inflammation/granulomas** | 0 | None |
|  | 1 | Minimal — One focus per 4× objective |
|  | 2 | Mild — Two to four foci per 4× objective |
|  | 3 | Moderate — Five to 10 foci per 4× objective |
|  | 4 | Marked — >10 foci per 4× objective |
| **Necrosis** | 0 | None |
|  | 1 | Minimal — one focus per 10× objective |
|  | 2 | Mild — Two to four foci per 10× objective |
|  | 3 | Moderate — Five to ten foci per 10× objective |
|  | 4 | Marked — >10 foci per 10× objective |

**Table 4. Histopathological scoring criteria for Lung**

| **Lung** | Score | Description |
| --- | --- | --- |
| **Granulomas** | 0 | None |
|  | 1 | Minimal—One focus per 4x objective |
|  | 2 | Mild—Two to four foci per 4x objective |
|  | 3 | Moderate—Five to 10 foci per 4x objective |
|  | 4 | Marked—>10 per 4x objective |
| **Neutrophilic accumulation** | 0 | None |
|  | 1 | Minimal—few cells identified |
|  | 2 | Mild—multiple small foci <10 cells |
|  | 3 | Moderate—1-2 foci of >10 cells |
|  | 4 | Marked—Multiple foci of >10 cells |
| **Necrosis** | 0 | None |
|  | 1 | Minimal—one focus per 10x objective |
|  | 2 | Mild—Two to four foci per 10x objective |
|  | 3 | Moderate—Five to ten foci per 10x objective |
|  | 4 | Marked—>10 foci per 10x objective |
| **BALT hyperplasia** | 0 | None |
|  | 1 | Present |
